# The Multipurpose Usage of Diffusion-Weighted MRI in Rectal Cancer

**DOI:** 10.3390/medicina59122162

**Published:** 2023-12-13

**Authors:** Aneta Yacheva, Dragomir Dardanov, Dora Zlatareva

**Affiliations:** 1 Department of Diagnostic Imaging, University Hospital Alexandrovska, Medical University of Sofia, 1431 Sofia, Bulgaria; 2 Department of Surgery, University Hospital Lozenetz, 1407 Sofia, Bulgaria

**Keywords:** MRI, rectal cancer, ADC, chemoradiotherapy

## Abstract

*Background and Objectives:* Colorectal cancer is the third most common oncological disease worldwide. The standard treatment of locally advanced rectal tumors is neoadjuvant radiochemotherapy in combination with surgical resection. The choice of specific treatment algorithm is highly dependent on MRI findings. The aim of this study is to show the potential role of ADC measurements in rectal cancer and their usage in different clinical scenarios. *Materials and Methods:* A total of 135 patients had rectal MRI evaluation. Seventy-five (56%) had histologically proven rectal adenocarcinoma and sixty (44%) were evaluated as rectal disease-free. An ADC measurement in the most prominent region of interest was obtained for all patients. Eighteen patients (24% of the rectal cancer group) had a second MRI after neoadjuvant chemoradiotherapy with comparison of the ADC values at the same region of interest as previously measured. *Results:* Rectal cancer ADC values were found to be significantly lower than the ones in the control group (*p* < 0.001). A statistically significant correlation was found when ADC values in rectal tumors of different T stages were compared (*p* = 0.039)—those with higher T stage as in locally advanced disease showed lower ADC values. Patients with extramural vascular invasion showed significantly lower ADC values (*p* = 0.01). There was a significant increase in ADC values after treatment (*p* < 0.001), and a negative correlation was observed (r = −0.6572; *p* = 0.004)—tumors with low initial ADC values showed a higher increase in ADC. *Conclusions:* ADC measurements have a complementary role in the assessment of rectal cancer and have the potential to predict the response to chemoradiotherapy and improve the planning of proper treatment strategies.

## 1. Introduction

Colorectal cancer is the third most common cancer in both men and women according to the International Agency for Research on Cancer Data published in 2023, with 35% of colorectal cancer cases in Europe occurring in the rectum [1]. In terms of cancer mortality, colorectal carcinoma ranks second in the world [2]. The standard treatment of locally advanced rectal tumors adopted by the American Society of Clinical Oncology (ASCO) is neoadjuvant radiochemotherapy in combination with surgical resection. Recently, total neoadjuvant treatment has shown promising results and has become an effective treatment option for patients with locally advanced rectal cancer according to the data from RAPIDO and PRODIGE23 trials [3,4].

With its high soft-tissue sensitivity and the use of various sequences, magnetic resonance imaging is the method of choice for the accurate staging of rectal cancer. MRI has a key role in determining the most appropriate therapeutic behavior. Preoperative assessment of tumor response is essential for planning the most appropriate treatment strategy. Evaluating the tumor response to therapy by MRI is of great value for optimizing surgical approaches. Diffusion-weighted imaging (DWI) is a functional non-invasive technique based on the free Brownian motion of water molecules in different tissues. DWI detects and highlights the differences in the mobility of water molecules in the extracellular space of biological tissues [5]. Hypercellular tissues such as tumors limit the diffusion of water molecules. DWI is known for its important role during the restaging of rectal cancer, mainly for its use in differentiating vital residual tumors from fibrosis [6]. The apparent diffusion coefficient (ADC) is derived from DWI and represents a calculated value that quantitatively reflects the restriction of diffusion. Low ADC values accurately give information on the restriction of the mobility of water molecules, which can be highly influenced by the presence of obstacles such as tumors of high cellular density [7]. There are several studies that suggest that measuring ADC values and comparing them after chemoradiotherapy (CRT) may be useful in predicting the tumor response to treatment [8,9,10,11]. The role of ADC measurement has been studied in tumors in other organs, such as the breast and prostate, and results in the literature have demonstrated the value of ADC for reflecting the biological features of various tumors and predicting tumor behavior and patient outcomes [12,13]. However, measuring ADC values is not yet accepted or recommended by the European Society of Gastrointestinal and Abdominal Radiology (ESGAR) for routine use in rectal cancer staging due to the lack of standardized protocols and validated thresholds [14]. There is still a lack of sufficient investigation data on the role of ADC as an important potential prognostic factor for tumor behavior and expected tumor response after treatment. Further research needs to be performed to better understand the potential utility of measuring ADC values in the planning of the proper and on-time management of patients with rectal cancer.

The aim of this study is to further investigate this issue and compare our results to those of previous studies, especially on a local level, and in this way, contribute to better demonstrating the potential role of ADC measurements in rectal cancer and its usage in different clinical scenarios.

## 2. Materials and Methods

Overall, 135 patients had rectal MRI evaluation. For 75 (56%) of the patients, the exam was a part of preoperative planning for previously known rectal cancer, while for the remaining 60 patients (44%), indications were non-neoplastic. All patient data were obtained retrospectively from our hospital database archives. All patients sign a consent form before they undergo an MRI exam stating that their data can be used for scientific or educational purposes.

MRI was performed on 75 patients (39 male, 36 female) with histologically verified adenocarcinoma of the rectum. The age range was 32–85 with a mean age of 66. Only patients with no history of other malignancies were included, as well as those who had not received chemoradiotherapy previously. Eighteen of those patients (24%) received preoperative neoadjuvant chemoradiotherapy, and a second follow-up MRI was performed after completion of the course to evaluate the response. Sixty patients with no rectal pathology were included as a control group.

Imaging was acquired using a 3 Tesla MRI scanner—Magnetom Verio, A Tim+ Dot System, Siemens, Germany. The imaging protocol included multiplanar T2-weighted sequences (sagittal, oblique axial and oblique coronal plane) with thin-section images in a small field of view (FOV). Oblique axial images were obtained perpendicular to the tumor and oblique coronal—parallel to the anal canal. Additional postcontrast T1 sequences were also included. DWI was performed with b-values of 50, 400 and 800 s/mm^2^, and a corresponding ADC map was created.

Staging was performed according to the latest 8th revision of the TNM Classification of Malignant Tumors by the American Joint Committee on Cancer (AJCC) adopted in 2016 [15]. Tumor response to CRT was assessed using the Response Evaluation Criteria in Solid Tumors (RECIST).

ADC measurements of the rectal wall were obtained for all patients using a single-slice 15–20 mm^2^ region of interest (ROI) and calculating an average of 3 focuses with the lowest signal. The mean ADC values of all patients were compared using the one-way ANOVA test and/or Pearson’s correlation test. Pretreatment ADC values were also compared to ADC values after completion of chemoradiotherapy in 18 patients who were followed-up.

A *p*-value less than or equal to 0.05 was considered statistically significant.

## 3. Results

Each exam was independently evaluated by two radiologists based on a blind review of the images, and ADC was measured in the most prominent region. Each patient’s region of interest (ROI) ADC value was stored as an image for comparison if a follow-up study was performed.

**Figure 1:**
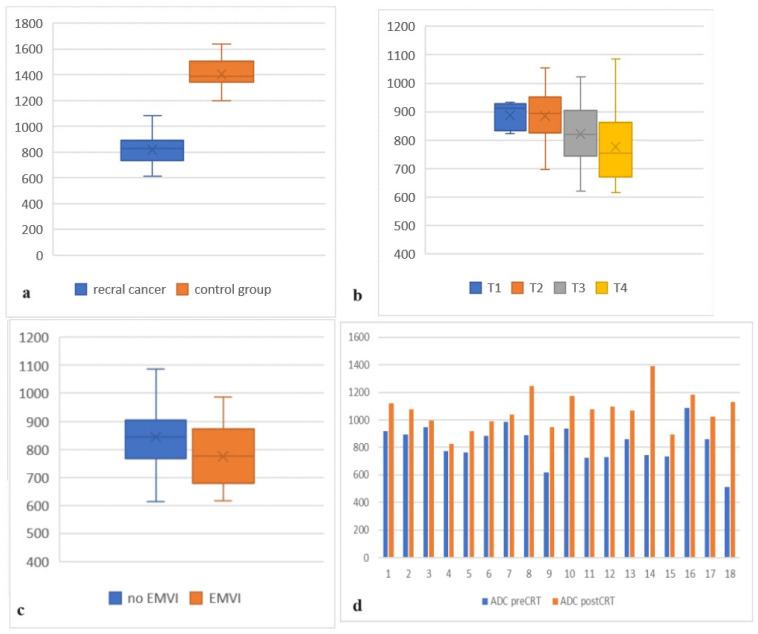
(**a**) ADC values of rectal cancer patients compared to ADC values of the control group; (**b**) comparison between ADC values in different T stage subgroups; (**c**) comparison between ADC values in patients with and without EMVI; (**d**) comparison between pretreatment ADC values and ADC values after CRT in 18 patients.

The initial pretreatment mean ADC value of all 75 patients with proven rectal cancer was 841 (min 615; max 1086). The mean ADC value of the normal rectal wall in the control group of 60 patients was 1405, which appeared to be significantly higher with a *p*-value of <0.001 (Figure 1a; Table 1).

**Figure 2:**
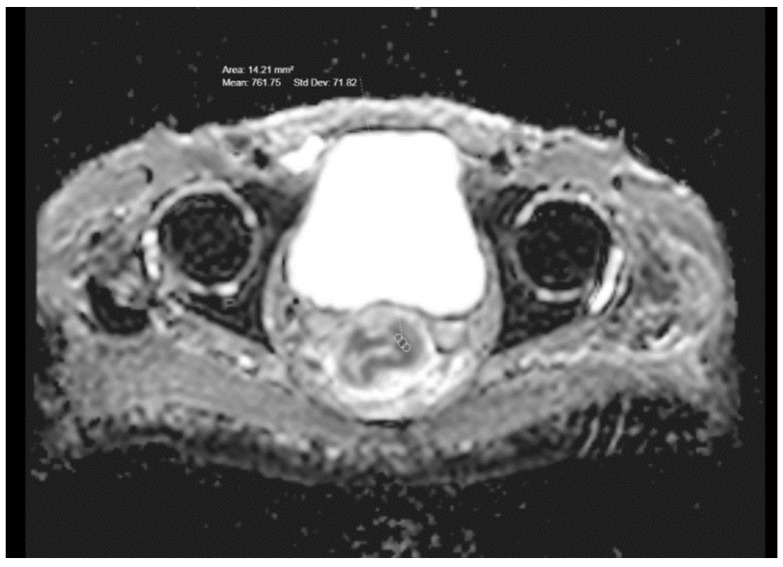
Pretreatment ADC value in a T3-stage rectal cancer.

A statistically significant correlation was found when the ADC values in the rectal tumors of different T stages were compared (*p* = 0.039)—those with higher T stages showed lower ADC values (Figure 1b and Figure 2; Table 1). The mean ADC values in T1-stage tumors (5 patients) and T2-stage tumors (14 patients) were highest at 887 and 881, and the mean ADC value in T3 (38 patients) was 819 and in T4 (18 patients) was 776. No significant correlation was found between the ADC values of T3- and T4-stage tumors (*p* = 0.36) but T3- and T4-stage tumors showed significantly lower ADC values when compared to T1- and T2-stage (*p* < 0.05).

The mean ADC value in cases with nodal involvement (38 patients) was 825, which showed no statistically significant correlation when compared to cases without nodal involvement (*p* = 0.5). The mean ADC value in cases with distant metastases (16 patients) was lower—819. However, these values also did not show a significant correlation when compared to cases with no presence of distant metastases (*p* = 0.4).

A statistically significant difference (*p* = 0.01) was found between the ADC values of 27 patients (36%) with extramural vascular invasion (EMVI) and patients without EMVI (Figure 1c; Table 1). The mean ADC value in patients with EMVI was overall lower (782) than the mean ADC of all 75 rectal cancer cases (841).

There was no significant correlation between ADC values and the histological degree of tumor differentiation (*p* = 0.32), although poorly differentiated tumors had overall lower ADC values—the mean ADC value in poorly differentiated tumors (8 patients) was 779, in moderately differentiated tumors (62 patients) was 854 and in well-differentiated tumors (5 patients) was 838.

**Figure 3:**
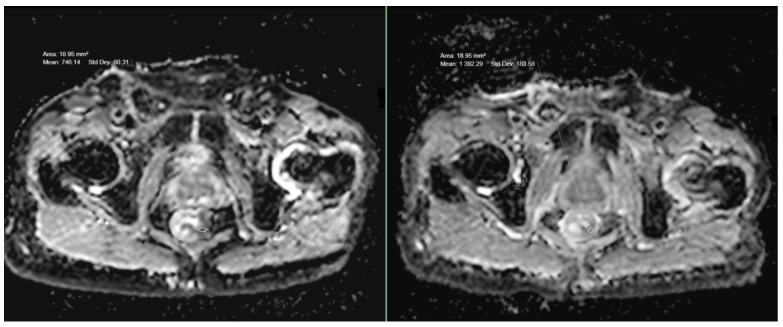
ADC values in a case of T3-stage rectal cancer before and after treatment.

A total of 14 (78%) of all 18 patients who underwent neoadjuvant chemoradiotherapy (CRT) showed a partial response in the follow-up MRI with a >30% reduction in the size of the tumor. In three patients (16%), there was no significant difference in the size of the tumor, and in one patient (5%), there was a progression in the size. However, an increase in the ADC values was observed in all 18 cases (Figure 1d and Figure 3; Table 2). The mean ADC value after CRT was 1067. The mean increase in the ADC values after CRT compared to the initial ADC values was 241, which was shown to be highly significant (*p* < 0.001). The mean increase in ADC values in T2-stage tumors was higher (257; *p* < 0.05) than in T1- (202; *p* < 0.05) and T3-stage tumors (205; *p* < 0.05). Locally advanced T4-stage tumors showed the highest increase in ADC values (288; *p* < 0.05).

**Figure 4:**
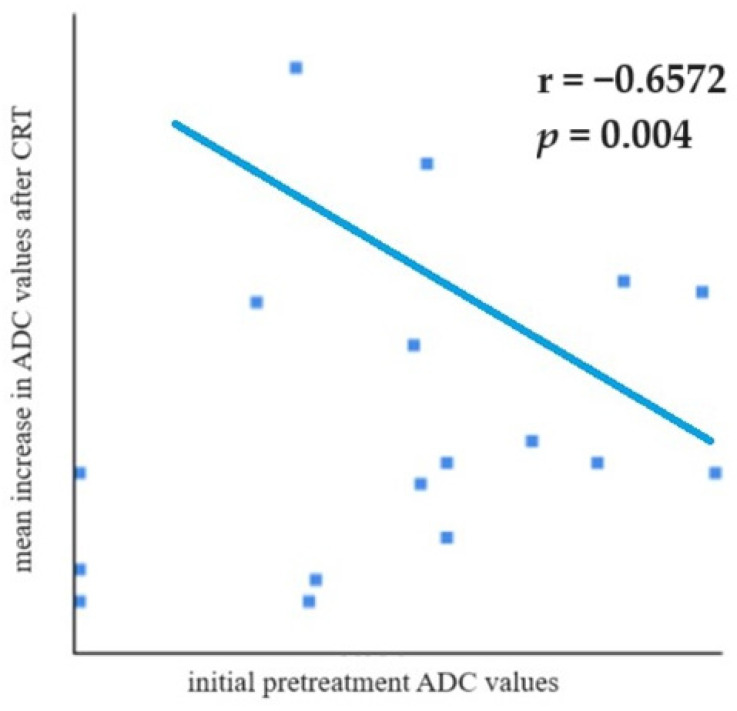
Pearson’s correlation test showed a negative correlation between initial pretreatment ADC values and the mean increase in ADC after CRT (r = −0.6572; *p* = 0.004).

Pearson’s correlation test was used to compare initial pretreatment ADC values and the mean increase in ADC values after treatment (Figure 4). A statistically significant and moderately strong negative correlation was observed (r = −0.6572; *p* = 0.004)—tumors with lower pretreatment ADC values showed a higher increase in ADC values after treatment, meaning a prominent response to therapy, or in other words, lower tumor cell differentiation is prone to high CRT sensitivity.

## 4. Discussion

MRI plays a key role in the primary staging of rectal cancer with postoperative restaging and monitoring for local recurrences or residual formations. Various sequences allow for accurate assessment of the depth of tumor invasion and its relation to surrounding organs, as well as for the identification of important prognostic factors that may alter the course of therapy. Neoadjuvant radiochemotherapy is applied in patients with locally advanced tumors in clinical stages T3 and T4 to reduce the size of the tumor in order to achieve clear resection margins (R0) and reduce the risk of local recurrences [16]. MRI is the modality of choice for restaging due to its high soft-tissue sensitivity compared to other modalities such as rectal ultrasound, CT or PET/CT [17]. MRI is of great value in evaluating the response to chemoradiotherapy and planning the time and type of further surgical treatment [18,19]. In a selected group of patients with complete response to chemoradiotherapy, when there are no signs of tumor presence, an organ-preserving “Watch-and-Wait” strategy can be applied [20]. Therefore, it is of great importance that proper preoperative restaging by using MRI be performed for the accurate assessment of tumor response to chemoradiotherapy and for the most appropriate treatment strategy to be obtained.

DWI is a non-invasive functional imaging technique based on the Brownian movement of water molecules and it is routinely used in rectal cancer staging protocols. DWI with high b values (≥800 s/mm^2^) is recommended by the European Society of Gastrointestinal and Abdominal Radiology (ESGAR) for routine examination, especially during restaging in order to qualitatively assess the response to neoadjuvant radiochemotherapy, as it has a key role in differentiating partial from complete responses [14,16]. This is especially true in terms of differentiating between post-treatment changes such as chemoradiotherapy-induced fibrosis and residual or recurrent vital tumor tissue, since high DWI signal intensity reflects the restriction of diffusion from tumors with high cellular density [6]. However, some pitfalls and difficulties exist in the interpretation of diffusion-weighted images like the “T2 shine-through” effect and sensitivity to susceptibility artifacts. ADC is a quantitative parameter derived from DWI that can be used to evaluate tissue cellularity and differentiate between benign and malignant lesions, although it has not yet been accepted in daily practice due to the lack of standardized protocols and validated thresholds [14]. Another potential problem in using ADC measurements in routine practice could be the misinterpretation of low ADC values in areas of post-treatment fibrosis as vital tumor tissue—in these cases, DWI will also demonstrate low signal intensity due to the presence of fibrosis [21,22]. Mucinous tumors, which have overall worse prognosis, have higher ADC values due to the high mucin component and low cellular density [23,24]. The lack of restriction of diffusion in these tumors could also be misinterpreted as a lack of malignancy and be a potential pitfall in using ADC measurements in daily practice. The aim of this study was to explore the relationships of ADC values in rectal cancer characteristics and the potential use of ADC according to different clinical situations.

When we compared the ADC values of normal rectal walls in the control group with the pretreatment ADC values of the proven rectal cancer patient group, a statistically significant decrease in the mean ADC values was observed in all T-stage subgroups. This demonstrates that low ADC values reflect tissue hypercellularity and are strongly associated with the presence of malignancy. Our results are consistent with those from other studies [5,25,26] and point out the role of ADC values in the differentiation between benign and malignant lesions.

Our study suggests that lower ADC values are strongly related to higher T stages. We found that tumors with high T stages have lower pretreatment ADC values, especially in locally advanced tumors, where a significant difference between T3/T4 stage and T1/T2 stage was found. This could be due to the fact that ADC is derived from the free movement of water molecules which can be influenced by high cellular density and heterogeneity [8]. Our results complement those of other studies [8,9,10] and demonstrate that pretreatment ADC values could potentially reflect the aggressiveness of tumors.

There was a trend in our study that tumors with nodal and/or distant metastases had lower ADC values compared to tumors of the N0 and M0 stages, although there was no statistically significant correlation. This could be due to the small exam group and the ROI measurement technique that we used.

We measured lower ADC values in patients with extramural vascular invasion compared to patients without EMVI, which were shown to be statistically significant. Extramural vascular invasion is defined as direct tumor invasion of perirectal veins and is often found upon histopathological exam. The presence of extramural vascular invasion is often associated with locally advanced rectal cancer of stage T3 and above since lower-stage tumors like T1 and T2 are contained only in the rectal wall and do not show infiltration beyond the muscularis propria. MRI can be used for the early detection of EMVI before histopathological exam is performed [27]. Extramural vascular invasion can be seen on T2-weighted images as nodular or serpiginous irregular structures of low signal intensity that originate from the primary tumor and can be observed following the course of perirectal veins [28]. MRI-detected EMVI has been recognized to be an important factor in the decision-making and planning of rectal cancer treatment [29,30]. Venous invasion is considered to be the first step to hematogenous dissemination [31]. It is proven that direct venous invasion from tumors that drain to the inferior mesenteric vein, such as rectal cancer, facilitates hematogenous spread through the portal vein circulation and most often leads to metastases in the liver [32,33]. Therefore, EMVI is often associated with the presence of distant metastases and local recurrences and is considered an independent prognostic factor of overall patient survival [27,34,35]. The presence of extramural vascular invasion can influence further treatment strategies and is often an indication for applying more aggressive neoadjuvant chemoradiotherapy [30,36]. Our results show significantly lower ADC values in tumors with the presence of EMVI on MRI compared to those where no EMVI was detected. The data from our study population are similar to the results of other studies that investigate ADC values in patients with and without EMVI [37,38]. These results could indicate that lower ADC values in rectal tumors can be linked to more aggressive tumor behavior and could be a predictor of presence of EMVI, and therefore, worse patient prognosis. This also demonstrates that adding diffusion-weighted sequences to standard MRI protocols that use mainly T2 sequences for tumor evaluation could be beneficial for the early detection of extramural vascular invasion. Therefore, DWI/ADC images could be crucial for decision making and further treatment planning for patients with rectal cancer.

The histological degree of tumor differentiation has also been shown to be an important prognostic factor. There are similar studies to ours that prove a significant correlation between ADC values and tumor differentiation grade [10,39]. However, in our study, we found no statistically significant correlation between ADC values and tumor differentiation grade, which could be due to the small study population in different histological subtypes, particularly in poorly and well-differentiated tumors.

We compared the pretreatment ADC values to the ADC values after the completion of chemoradiotherapy. A total of 14 of 18 patients that were followed-up showed regression in the tumor size on the follow-up MRI, and the mean increase in the ADC values was statistically significant, which correlates with a good therapeutic response. A decrease in tissue cellularity and an increase in extracellular space after CRT leads to an increase in ADC values [11,40]. Because of cellular damage and loss of membrane integrity due to chemoradiotherapy, the mobility of water molecules in the tissue microenvironment increases, and therefore, ADC values also increase [41]. Measuring the size of tumors is a technique that is widely used as a marker of tumor response and assessment of the degree of tumor regression after the preoperative neoadjuvant chemoradiotherapy course is finished so that a proper surgical approach can be applied for further patient treatment. According to the RECIST criteria which are commonly used for the evaluation of tumor response in solid tumors, a reduction of at least 30% in the sum of the longest diameters of the tumor is considered a partial response, and an increase of 20% is considered disease progression [42]. Although not all patients in our study population showed regression in tumor size after therapy, all 18 patients showed an increase in ADC values, which suggests that an increase in ADC can be observed before a reduction in the size of the tumor occurs. This could indicate that DWI/ADC can be used as an earlier prognostic factor for tumor response to treatment prior to tumor size measurements. Such observations which state that changes in ADC measurements often precede changes in tumor size have been made by other authors, as well, and they suggest that response evaluation by using ADC might influence clinical practice by allowing for proper treatment adjustments for optimizing the management of rectal cancer patients [41].

In our study, the mean increase in ADC values was highest in T4-stage tumors. We found a statistically significant and moderately strong negative correlation between pretreatment ADC values and the mean increase in ADC after CRT—locally advanced tumors with lower pretreatment ADC values showed a higher increase in ADC values after treatment, and those with high initial values did not increase that much. Our results complement those of other studies [43,44], which suggests that measuring ADC values as part of a routine rectal MRI exam could be beneficial for predicting treatment response, as tumors with low initial ADC values are more likely to respond better to CRT. This could be due to the fact that tumors with higher pretreatment ADC values are more likely to have areas of necrosis, which is associated with poor tissue perfusion and higher resistance to radiochemotherapy [45]. Our results suggest that measuring ADC values can be of help in proper decision making for patient treatment—in patients with tumors with high ADC values and expected poor response, adverse effects from CRT can be avoided and decisions for surgery can be taken earlier. Adding ADC measurements to standard MRI protocols used for the staging of rectal cancer seems to have the potential to improve patient management, optimize further therapy and increase the chances for better treatment outcomes.

Artificial intelligence (AI) is continuously and rapidly developing in the medical field and shows potential use in medical diagnosis, treatment planning and patient prognosis, including in the area of rectal cancer management [46]. There are several studies that demonstrate the potential application of AI in the prognosis of rectal cancer’s response to treatment using different models based on DWI images and ADC maps [47,48,49]. Large-scale analyses could be performed using these models to further investigate the future perspectives of using ADC values in rectal cancer evaluation.

Our study has several limitations. First, our study consists of a relatively small group of patients, particularly those that were followed-up with MRI after CRT, and further research needs to be performed to validate the prognostic utility of ADC values. The second limitation is the ROI measurement technique that was used—we obtained three single-slice ROI samples for ADC measurements, which might not be fully representative of the entire tumor volume and characteristics.

## 5. Conclusions

MRI is known to have a key role in rectal cancer staging. Although not yet accepted in routine protocols, ADC measurements are an effective and convenient addition to traditional imaging methods. Our study suggests that they may have a significant diagnostic value and a complementary role in the assessment of rectal cancer patients and in further optimizing patient management. Measuring ADC values is a non-invasive technique that correlates with important tumor features. ADC has the potential to become a predictor of tumor behavior and the prognosis of tumor response to chemoradiotherapy, and can help in the improvement of proper treatment strategy planning. Selecting patients who are more likely to respond better to CRT based on their ADC values can prevent risks related to delayed surgery and unnecessary toxicity.

## Figures and Tables

**Table 1:** Comparison between mean ADC values in different rectal cancer subgroups.

		Mean ADC	*n*	%	*p*-Value
control group		1405	75	56%	<0.001
rectal cancer group		841	60	44%	
T-stage	T1	887	5	6.7%	0.039
	T2	881	14	18.7%	
	T3	819	38	50.7%	
	T4	776	18	24%	
patients with nodal involvement		825	38	51%	0.5
patients with distant metastases		819	16	21%	0.4
patients with EMVI		782	27	36%	0.01
histological degree of tumor differentiation	poorly differentiated	779	8	11%	0.32
	moderately differentiated	854	62	83%	
	well differentiated	838	5	7%	
post-CRT group		1154	18	24%	<0.001

**Table 2:** Comparison between the mean increase in ADC values after CRT in 18 patients.

	Mean Increase in ADC Values after CRT	% Mean Increase	*p*-Value
all	241	29%	<0.05
T1	202	22%	<0.05
T2	257	30%	<0.05
T3	205	25%	<0.05
T4	288	37%	<0.05

## Data Availability

The data presented in this study are available on request from the corresponding author.
